# The maternal energetic environment affects both egg and offspring phenotypes in green anole lizards (*Anolis carolinensis*)

**DOI:** 10.1002/ece3.9656

**Published:** 2023-01-06

**Authors:** Jamie R. Marks, Mahaut Sorlin, Simon P. Lailvaux

**Affiliations:** ^1^ Department of Biology University of New Orleans New Orleans Louisiana USA

**Keywords:** *Anolis carolinensis*, diet manipulation, maternal effects, sprint training

## Abstract

Animals exist in dynamic environments that may affect both their own fitness and that of their offspring. Maternal effects might allow mothers to prepare their offspring for the environment in which they will be born via several mechanisms, not all of which are well understood. Resource scarcity and forced resource allocation are two scenarios that could affect maternal investment by altering the amount and type of resources available for investment in offspring, albeit in potentially different ways. We tested the hypothesis that maternal dietary restriction and sprint training have different consequences for the offspring phenotype in an oviparous lizard (*Anolis carolinensis*). To do this, we collected and reared eggs from adult diet‐manipulated females (low‐diet [LD] or high‐diet [HD]) and sprint‐trained females (sprint trained [ST] or untrained [UT]) and measured both egg characteristics and hatchling morphology. ST and LD mothers laid both the fewest and heaviest eggs, and ST, UT, and LD eggs also had significantly longer incubation periods than the HD group. Hatchlings from the diet experiment (LD and HD offspring) were the heaviest overall. Furthermore, both body mass of the mother at oviposition and change in maternal body mass over the course of the experiment had significant and sometimes different effects on egg and offspring phenotypes, highlighting the importance of maternal energetic state to the allocation of maternal resources.

## Graphical abstract

We manipulated diet (low quantity of food or high quantity of food) and energetic output (sprint trained or untrained) in green anole (*Anolis carolinensis*) females to test how variation in the maternal environment affects offspring phenotype. We learned that these different environments caused different phenotypic effects, such as affecting egg mass, incubation period, and hatchling mass. These results demonstrate that the maternal energetic environment (i.e., the amount of available energy to the mother) has significant effects on both the egg and offspring phenotypes.

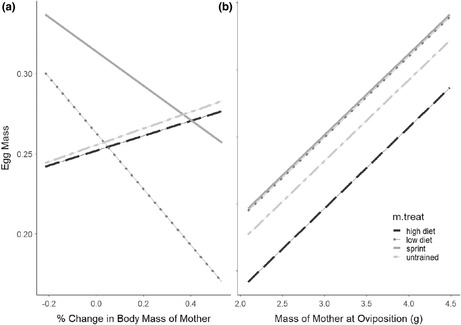

## 1 INTRODUCTION

An animal's environment is constantly changing, with many taxa facing variable temperatures, changes in resource availability, or changes in predator presence over relatively short timescales. Phenotypic plasticity might ameliorate the fit between individual and environment (Ghalambor et al., 2007; Losos et al., 2000), but females can also influence offspring phenotypes and fitness via maternal effects, defined as the phenomenon whereby the offspring phenotype can be affected by the environment that the mother experiences (Wolf & Wade, 2009). Maternal effects can manifest directly as alterations in sex ratios (Mousseau & Fox, 1998), brood size (Brown & Shine, 2009; Stearns, 1989), or hatchling size (Brown & Shine, 2009; Sinervo & Huey, 1990; Stearns, 1989) among other effects (Ensminger et al., 2018). But although female plasticity is well documented, we lack an understanding of how female plastic responses to specific types of environmental variation affect offspring resource allocation, and ultimately, offspring phenotype. Causal variation driving these maternal effects is typically labeled broadly as “stress” or “environmental quality” which gives little insight into the factors driving such effects, or into the underlying mechanisms underlying them (Boots & Roberts, 2012; Glavin, 1984; Peixoto et al., 2020). Understanding these mechanisms is necessary for uncovering the functional links among life‐history trade‐offs (Stearns, 1989), transgenerational effects, and phenotypic variation (Bonduriansky & Day, 2020).

A mechanism that is known to drive trade‐offs in nearly all animals, specifically, the trade‐off between survival and reproduction, is diet restriction (Chapman & Partridge, 1996; Mair & Dillin, 2008; Moatt et al., 2016; Regan et al., 2020). Limiting resource acquisition can affect maternal provisioning, and thus drive maternal effects on offspring phenotypes. Oviparous females in particular provide insight into the maternal strategies employed in the face of different environmental pressures because mothers must proactively provision their eggs for the current environment (Giron & Casas, 2003; Romano et al., 2008; Saino et al., 2006). In addition to the phenotypes of the offspring themselves, maternal effects can also affect characteristics of the eggs, including their size, shape, and incubation periods (Dzialowski & Sotherland, 2004). For example, Madagascar ground geckos (*Paroedura picta*) under limited resource conditions not only exhibit longer periods between laying eggs, but those eggs are also smaller than those of well‐fed lizards (Kubička & Kratochvíl, 2009). Egg size also correlates with hatchling size, such that the resource‐limited females produced smaller juveniles (Kubička & Kratochvíl, 2009). Although the effects of restricted maternal diet on offspring phenotypes are well documented, the varying effects of different maternal environmental conditions on both egg and offspring phenotypes in vertebrates are poorly understood.

In addition to resource limitation, changes in environmental conditions can also drive crucial allocation trade‐offs in females, which could in turn affect the amount and type of resources available for mothers to allocate toward offspring. For instance, energetic investment into performance‐related traits such as predator evasion, foraging, and sprinting can also lead to changes in maternal phenotype which can in turn affect offspring phenotypes (Bro‐Jørgensen, 2013; Sheriff & Love, 2013; St‐Cyr et al., 2017). Increased activity or use of locomotor capacities, such as sprinting, can force an animal to invest energy into the underlying morphological and physiological mechanisms supporting that function, which can in turn promote trade‐offs (Husak & Lailvaux, 2019; Irschick et al., 2008; Lailvaux & Husak, 2014). In green anoles, sprint training was shown to increase both overall muscle size and investment in slow oxidative muscle fibers (Husak et al., 2015). Investment in muscle is especially costly, and likely incurs significant production and maintenance costs (Husak & Lailvaux, 2017). Because investment in locomotion can be easily manipulated in the laboratory through the implementation of specialized training regimes, this presents a useful opportunity to understand the effects of forced maternal allocation to an ecologically relevant trait.

In this experiment, we used green anole females (*Anolis carolinensis*), which are continuous reproducers (Love & Williams, 2008; Sparkman et al., 2010), to test the relative effects of maternal resource limitation and forced maternal resource allocation to locomotor capacity on both egg and offspring phenotypes. Continuous reproducers have incessant ovarian cycles and can store sperm and produce single‐egg clutches throughout the breeding season (Awruch, 2015; Lovern et al., 2004). We tested the hypothesis that maternal dietary restriction and maternal investment into sprint training would differently affect offspring phenotype. We made five specific predictions to test this hypothesis: (P1) the low‐diet (LD) and sprint‐trained (ST) animals would lay significantly fewer eggs than the high‐diet (HD) and untrained (UT) lizards; (P2) eggs and (P3) offspring from the LD and ST lizards would weigh less than those from the UT and HD moms; (P4) treatment would not affect SVL; and (P5) the incubation period for the treatment groups would be longer than that of their control counterparts.

## 2 MATERIALS AND METHODS

The eggs and offspring used in this experiment were derived from two prior experiments aimed at understanding how environmental variation, namely decreased resource acquisition (Marks et al., 2021) and increased investment in locomotion (Marks et al., 2022), affects the maternal phenotype. For continuity purposes, we chose to label our control groups based on their titles within the two previous manuscripts. The control group within the diet experiment is labeled high‐diet (HD) and the control group from the sprint experiment is labeled untrained (UT).

The UNO Institutional Animal Use and Care Committee protocol #19‐003 permitted all procedures outlined below. We captured adult, reproductively mature (snout–vent length (SVL) > 40 mm) *A. carolinensis* females from urban populations in Orleans Parish in Louisiana in June 2019 (*N* = 100) and June 2020 (*N* = 100), during the green anole breeding season (Jenssen et al., 1995). We recorded SVL to the nearest 0.05 mm and body mass to the nearest 0.01 g on the day of capture. The adult lizards were acclimated for 1 week prior to either treatment. The mothers were housed individually in a climate‐controlled room set to ~27°C.

### 2.1 Diet treatments

In June 2019, we tested the effects of energetic environment on insulin‐like growth factor expression in wild‐caught female green anoles by randomly allocating them to either a high‐diet (HD) or low‐diet (LD) group. Following the treatment protocol in Marks et al. (2021), all lizards were given ~1.25 cm crickets (*Acheta domesticus*). The LD group was fed one cricket coated in ZooMed ReptiCalcium powder, three times weekly, which is an established diet known to promote trade‐offs, whereas the HD group females were fed an ad libitum diet of three crickets, three times per week supplemented with ZooMed ReptiCalcium powder (as in Husak et al., 2016; Lailvaux et al., 2012). The HD “treatment” is therefore equivalent to the control situation, although we refer to these groups here as LD and HD to be consistent with Marks et al. (2021). The LD group was effective in decreasing reproductive output, consistent with Husak et al. (2016).

### 2.2 Sprint training

In June 2020, wild‐caught adult female lizards were randomly allocated to the untrained (UT) group or the sprint‐trained (ST) group. Both treatments were fed the same as the HD group in the previous experiment, which again corresponds to a “normal” or control diet. The ST group was trained following previously established protocol (Husak & Lailvaux, 2019; Marks et al., 2022; Wang & Husak, 2020). The ST lizards were sprint‐trained three times a week for 6 weeks. On each training day, they were encouraged to run up the dowel of a racetrack four times with each trial separated by at least 1 h. Training intensity was increased at weeks 2 and 4 by hanging weights off of the lizard equivalent to 25% and 50%, respectively, of the lizard's weekly body mass, as in Husak and Lailvaux (2019) and Wang and Husak (2020). The UT lizards were handled for 30 s three times a week to mitigate any stress effects due to the increase in handling time experienced by the ST animals (Husak et al., 2015). As for the diet treatment, we use the UT and ST labels for consistency with the earlier study (here Marks et al., 2022), but we note that the UT treatment corresponds to the control situation in sprint training studies (Husak et al., 2015; Husak & Lailvaux, 2019; Lailvaux et al., 2020).

### 2.3 Egg and hatchling husbandry

Egg collection began following the 1‐week acclimation period. Terraria were checked three times weekly for eggs by lightly sifting through the soil substrate on the bottom of the lizard terrarium. Dead and/or unfertilized eggs were recorded (i.e., date laid and maternal identification) and discarded. When an egg was found, it was placed on a digital scale and weighed to the nearest 0.01 g. Its length and width were also recorded with Mitutoyo digital calipers to the nearest 0.05 mm. Once morphometric measurements were taken, the egg was placed in a Petri dish with moist vermiculate. Eggs were individually held in Petri dishes and were labeled with the date they were found as well as maternal ID and were given a unique egg ID. The Petri dish was then placed in an incubator set to 28.6°C (Lovern et al., 2004; Lovern & Wade, 2003). Eggs in the incubator were watered gently with a spray bottle every other day and were rotated weekly to avoid position effects within the incubator. Eggs were checked daily for hatchlings.

When an egg hatched, the Petri dish was removed from the incubator and the hatchling was immediately weighed to the nearest 0.01 g and then housed in a terrarium under the same conditions as the adult females for future experiments. Offspring born in the 2020 sprint training experiment had their SVL measured to the nearest 0.05 mm with a Mitutoyo digital caliper on the same day they were removed from the incubator.

In short, we recorded the total number of eggs laid by each individual female and the total number of incubation days from oviposition to hatching. We also measured mass of the egg, initial mass at hatching, as well as the snout–vent length of the hatchlings from the sprint training experiment. Hatchling SVL was not recorded for the diet experiment due to unforeseen logistical challenges, and so we only present and analyze hatchling SVL for the sprint experiment here.

### 2.4 Statistical analyses

We used R version 3.6.0 (R Core Team, 2019) for all analyses. All models used maternal treatment (diet or sprint training or control group (HD and UT)) as a fixed factor. We tested for an effect of maternal mass as a covariate on each of the main dependent variables in two different ways. First, we included maternal body mass as a covariate because maternal body mass affects aspects of maternal physiology (see Marks et al., 2022, 2021) and is known to influence offspring phenotype (Shine & Downes, 1999; Warner & Lovern, 2014). Second, we included percent change in maternal mass over the course of the experiment calculated from the initial mass and final body mass measured (denoted here as %Δm.mass) as covariate as in Marks et al. (2022). We fit separate models for each covariate, such that models contained either maternal body mass or %Δm.mass, but not both. Therefore, we fit two saturated models for each dependent variable that differed only in the nature of the covariate, but that were otherwise identical.

For mixed models, all saturated models contained maternal identification nested within year of the experiment as a random factor to control for non‐independence of eggs from the same mother, and for year‐to‐year variation that might otherwise confound our results. We performed log‐likelihood deletion tests using the *MASS* package (Silk et al., 2020) to find minimum adequate models (i.e., the simplest models that explained the most amount of variation (Crawley, 1993) in all cases).

#### 2.4.1 Total number of eggs laid

We used the *glmer* command from the *lme4* package to fit a generalized linear mixed effects model with a Poisson distribution to test our first prediction (P1) that the number of eggs laid across treatments will be different. To visualize the model, we used packages *emmeans* and *ggplot2* to plot the treatment residuals after accounting for the effects of model covariates (as in Marks et al., 2021, 2022).

#### 2.4.2 Egg mass

To test P2, we used the *nlme* package to fit linear mixed‐effect models with our most saturated models containing %Δm.mass or maternal mass at oviposition as covariates. Maternal identification was again nested within year as a random effect. Maternal mass at oviposition told us the energetic state of the mother when the egg was laid while %Δm.mass told us the change in energetic state over the course of the experiment. To visualize, we used packages *emmeans*, *ggplot2*, and *gridExtra*.

#### 2.4.3 Hatchling mass

We used the *nlme* package to test our third prediction (P3) that maternal treatment affects mass of offspring at time of hatching. The saturated model contained the following covariates: egg mass, number of days in incubator, mass of the mother at oviposition, and %Δm.mass. To visualize the model, we generated a boxplot from *ggplot2* and used the *rstatix* package to overlay p‐values from a pairwise t‐test using a false discovery rate.

#### 2.4.4 SVL of hatchlings

We did not obtain SVL measurements at hatching from the 2019 diet experiment. However, we present the results from the 2020 sprint experiment to highlight the fact that the sprint training affected SVL of the offspring. To test our fourth prediction (P4) that sprint training affects offspring phenotype, we ran a linear mixed effects model with maternal identification as a random factor and included the following covariates to test if they affected SVL of the hatchlings: egg mass, number of days in incubator, mass of the mother at oviposition, and %Δm.mass. We visualized the data using *ggplot2* and used the package *rstatix* to overlay p‐values from a pairwise t‐test using a false discovery rate (García, 2003, 2004).

#### 2.4.5 Total incubation time

We used the *lme4* package in R (Bates et al., 2015) to fit an initial generalized linear mixed model with Poisson errors and maternal identity (because mothers produced multiple eggs) nested within year (i.e., 2019 or 2020) as random factors to test our fifth prediction (P5) that maternal energetic environment affects total incubation time of offspring and to deal with any year‐to‐year variation in these data. However, the model fit was not improved by the inclusion of any random factors; consequently, we fit a generalized linear model with only fixed factors to the incubation time data. To deal with underdispersion in the resulting model indicated by a dispersion factor (i.e., the ratio of residual deviance to degrees of freedom) <1, we fit a quasi‐Poisson distribution to the final minimum adequate model, which included an effect of %Δm.mass on incubation time. We used packages *emmeans* and *ggplot2* to visualize the final model by plotting the partial residuals. A partial residual is the distance between the predicted value and our data point when additional covariates are controlled for in the model (Cook, 1993).

## 3 RESULTS

### 3.1 Total number of eggs laid

**FIGURE 1:**
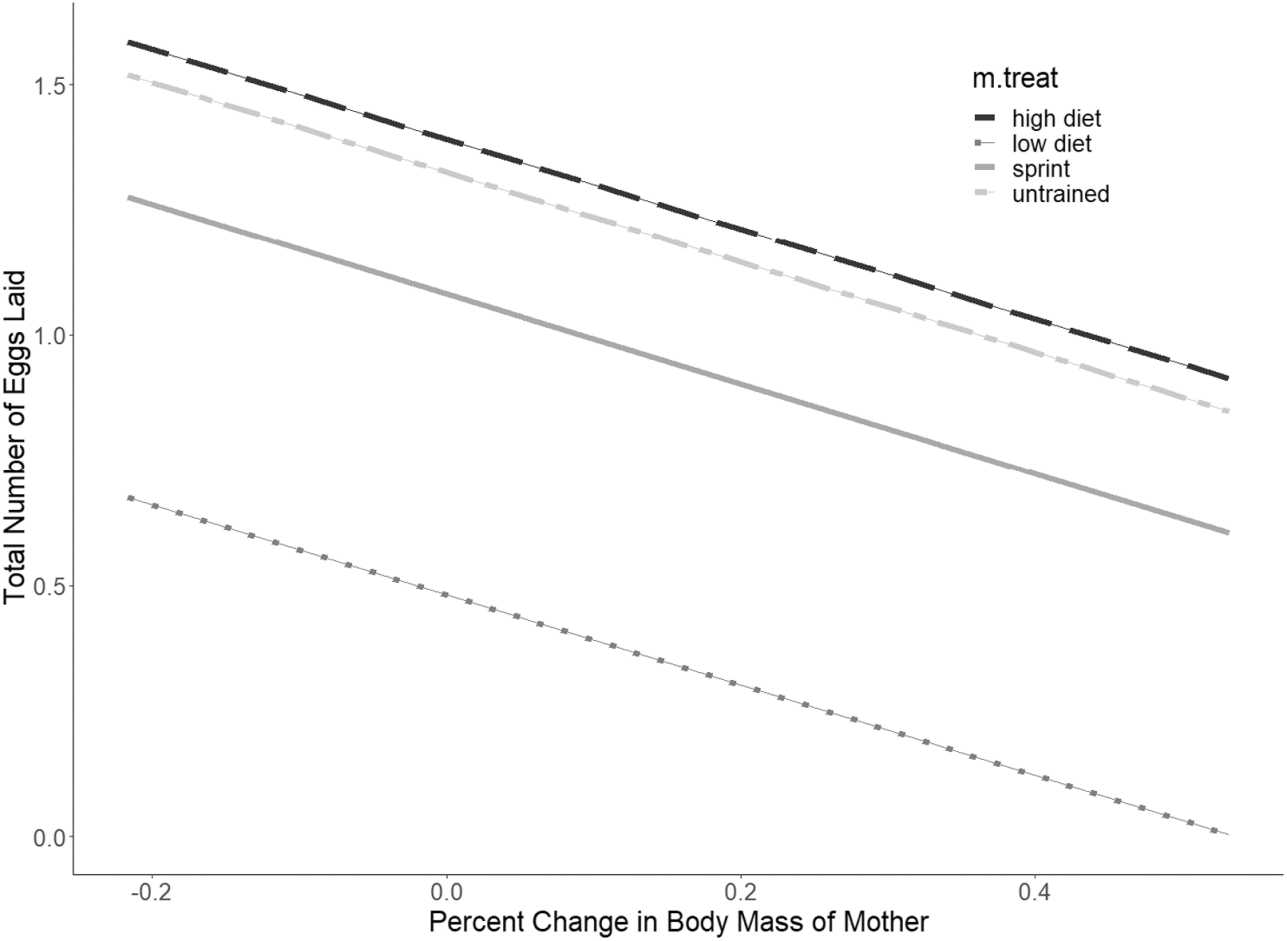
Graphs of the estimated marginal means for total number of eggs laid when percent change in body mass of the mother is accounted for.

Although the fit of our Poisson model to the egg number data was not ideal, fitting a negative binomial distribution returned qualitatively the same results, suggesting that our results are robust to distributional assumptions (Schielzeth et al., 2020; see also Warton et al., 2016, for discussion of distributional assumptions in count data). The model which best described how maternal treatment affects egg number was the model containing an effect of percent change in body mass of the mother (AIC = 1191.12, no. parameters = 2), followed by the model containing an interaction between treatment and percent change in body mass (AIC = 1193.33, no. parameters = 3). All treatments displayed a negative correlation between the total number of eggs laid and %Δm.mass (Figure 1; Table 1). Both the LD and ST groups laid significantly fewer eggs compared to the HD lizards, although egg numbers did not differ significantly between the HD and UT control groups. Maternal identification was included in the final model as a significant random effect.

**TABLE 1:** Best‐fitting models describing the variation in total number of eggs laid with %Δm.mass as a covariate.

Model term	Coefficient	SE
Intercept	1.39	0.11
Treat (LD)	−0.91	0.18
Treat (ST)	−0.31	0.12
Treat (UT)	−0.064	0.10
%Δm.mass	−0.90	0.37

*Note*: The reported coefficients give estimated change in the dependent variable between the baseline category and the categories named in the table (ST = sprint trained, UT = untrained, LD = low diet). Baseline category was the high‐diet group.

### 3.2 Egg mass

**FIGURE 2:**
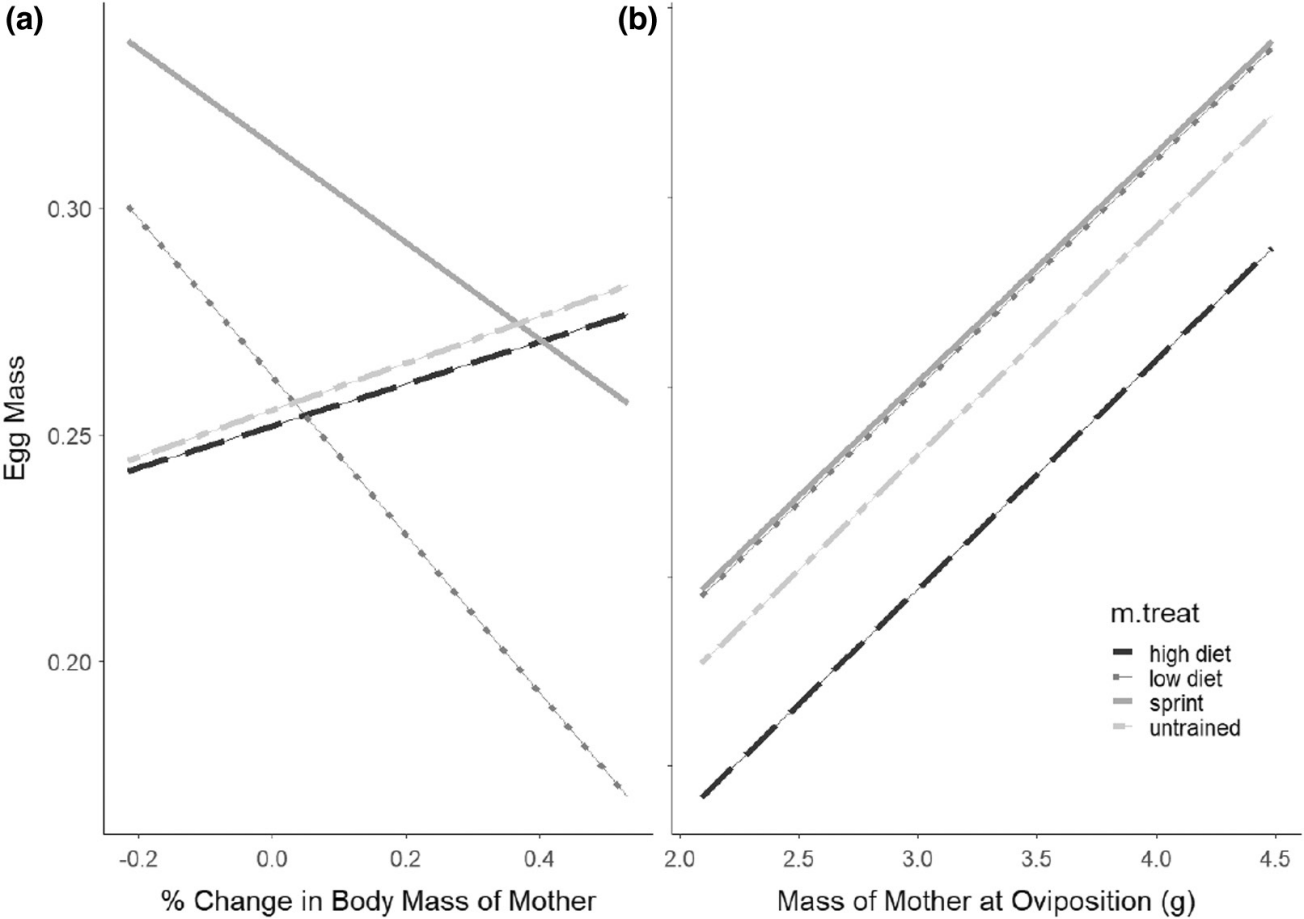
Graphs of the estimated marginal means for offspring egg mass when (a) percent change in body mass of the mother and (b) mass of the mother at oviposition are accounted for.

The model which best described how maternal treatment and energetic state over time affects egg mass was the model that retained an interaction between percent change in body mass of the mother and treatment (AIC = −654, no. parameters = 3) such that ST and LD lizards that gained mass over the course of the experiment laid lighter eggs than similarly sized UT and HD lizards (Figure 2a; Table 2). The next best model contained an effect of percent change in body mass on egg mass (AIC = −649, no. parameters = 2). ST and LD lizards that lost mass over the course of the experiment laid heavier eggs than their control counterparts. When looking at the model with mass of the mother at oviposition (Figure 2b; Table 3), the best model retained an effect of maternal mass at oviposition (AIC = −686, no. parameters = 2), which was positively correlated with egg mass, regardless of treatment. The next best model retained an interaction between mass of the mother at oviposition and treatment (AIC = −680, no. parameters = 3).

**TABLE 2:** Best‐fitting models describing the variation in egg mass with percent change in final body mass of the mom as a covariate.

Model term	Coefficient	SE
Intercept	0.25	0.015
Treat (LD)	0.0042	0.019
Treat (ST)	0.065	0.034
Treat (UT)	0.0029	0.023
%*Δ* mass	0.052	0.045
Treat (LD): %Δm.mass	−0.31	0.16
Treat (ST): %Δm.mass	−0.17	0.10
Treat (UT): %Δm.mass	0.0073	0.063

*Note*: The reported coefficients give estimated change in the dependent variable between the baseline category and the categories named in the table (ST = sprint trained, UT = untrained, LD = low diet). Baseline category was the high‐diet group.

**TABLE 3:** Best‐fitting models describing the variation in egg mass with mass at oviposition of the mom (m.massovi) as a covariate.

Model term	Coefficient	SE
Intercept	0.19	0.026
Treat (LD)	0.021	0.011
Treat (ST)	0.022	0.018
Treat (UT)	0.014	0.018
m.massovi	0.024	0.007

*Note*: The reported coefficients give estimated change in the dependent variable between the baseline category and the categories named in the table (ST = sprint trained, UT = untrained, LD = low diet). Baseline category was the high‐diet group.

### 3.3 Hatchling mass

**FIGURE 3:**
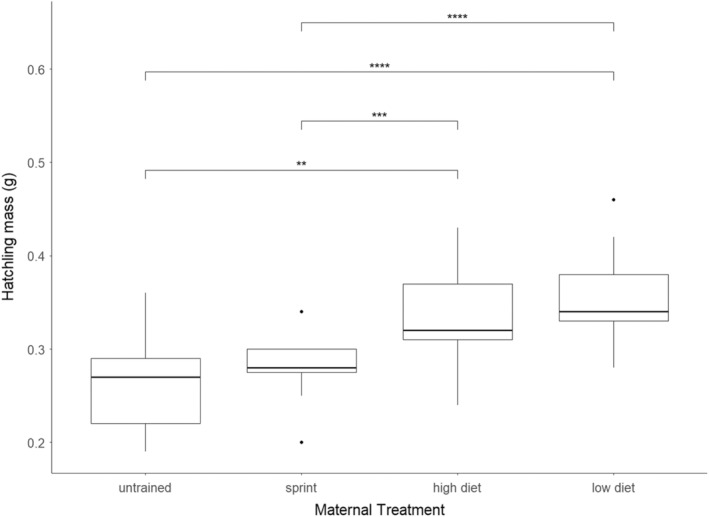
Boxplot showing the average mass of offspring at hatching. *p*‐Values from a false discovery rate pairwise *t*‐test are shown above plots (.001 > ***; .004 > **).

Our minimum adequate model did not contain any covariates (AIC = −302, no. parameters = 1), but maternal treatment did affect the mass at hatching. The next best model contained an effect of percent change in mass of the mother (AIC = −281, no. parameters = 2). Maternal identification was also nested within year as a random effect. The mass of the hatchlings from the UT and ST lizards averaged significantly less than the HD and LD lizards (Figure 3; Table 4). There was no significant difference in hatchling mass between the UT and ST or between the HD and LD lizards (note that year was included as a random factor in this analysis and thus accounted for).

**TABLE 4:** Best‐fitting models describing the variation in hatch mass among the four treatments.

Model term	Coefficient	SE
Intercept	0.33	0.007
Treat (LD)	0.017	0.014
Treat (ST)	−0.050	0.016
Treat (UT)	−0.066	0.016

*Note*: The reported coefficients give estimated change in the dependent variable between the baseline category and the categories named in the table (ST = sprint trained, UT = untrained, LD = low diet). Baseline category was the high‐diet group.

### 3.4 SVL of hatchlings

Our final model did not include any covariates (AIC = 130, no. parameters = 1), but maternal treatment did affect SVL at hatching. The following best model contained an effect of percent change in body mass on hatchling mass (AIC = 132, no. parameters = 2). Offspring from ST mothers were significantly longer than offspring from UT individuals, exhibiting significantly larger SVLs at hatching (Figure 5; Table 6).

**FIGURE 4:**
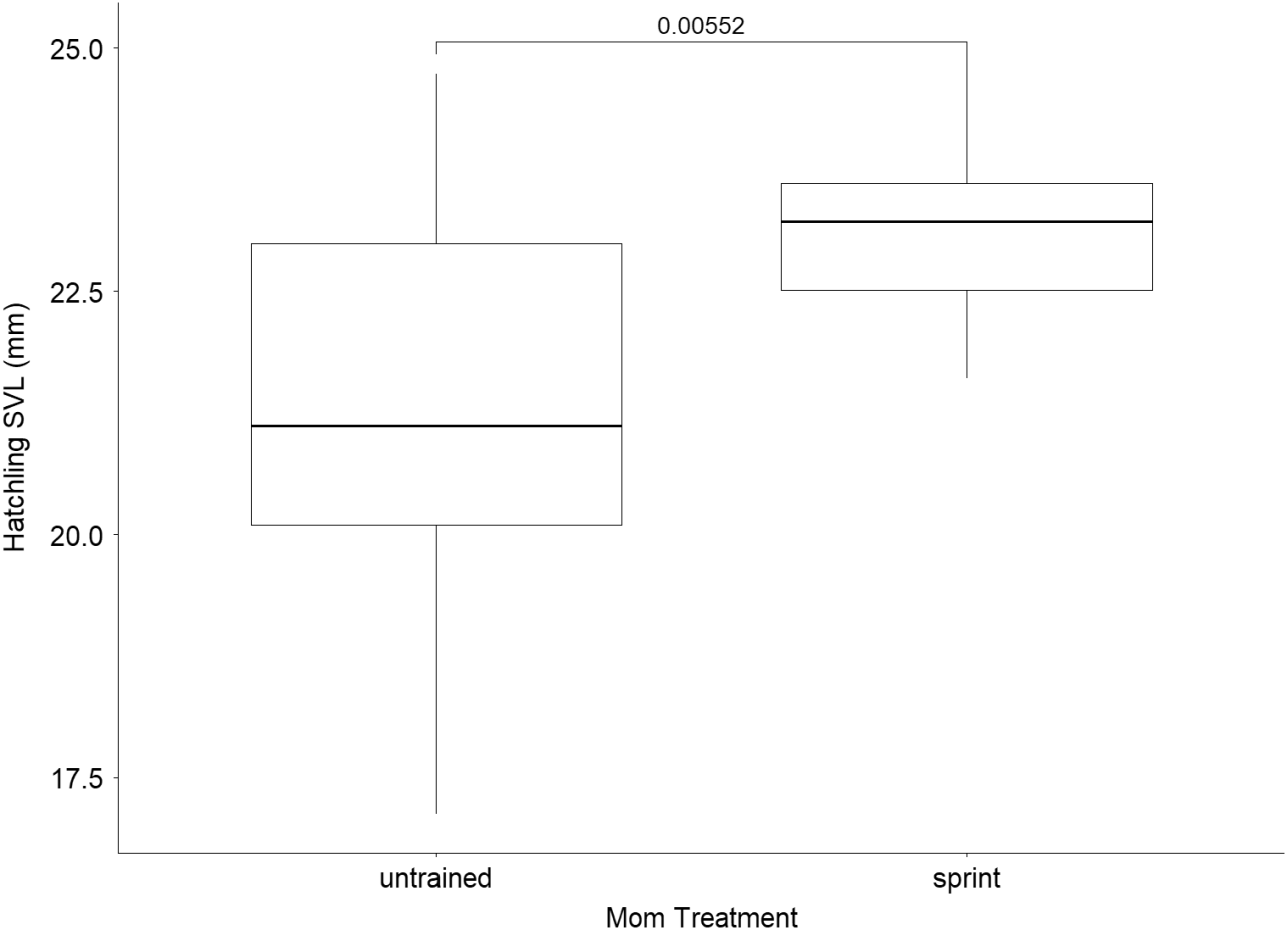
Boxplot showing the average SVL of offspring at hatching. *p*‐Values generated from a false discovery rate pairwise *t*‐test are shown above plots. Data for SVL at hatching from the 2019 diet experiment were not obtained.

**TABLE 5:** Best‐fitting model describing the variation in hatchling SVL.

Model term	Coefficient	SE
Intercept	23.13	0.30
Treat (UT)	−1.84	0.50

*Note*: The reported coefficients give estimated change in the dependent variable between the baseline category and the category named in the table (UT = untrained). Baseline category was sprint‐trained group.

**FIGURE 5:**
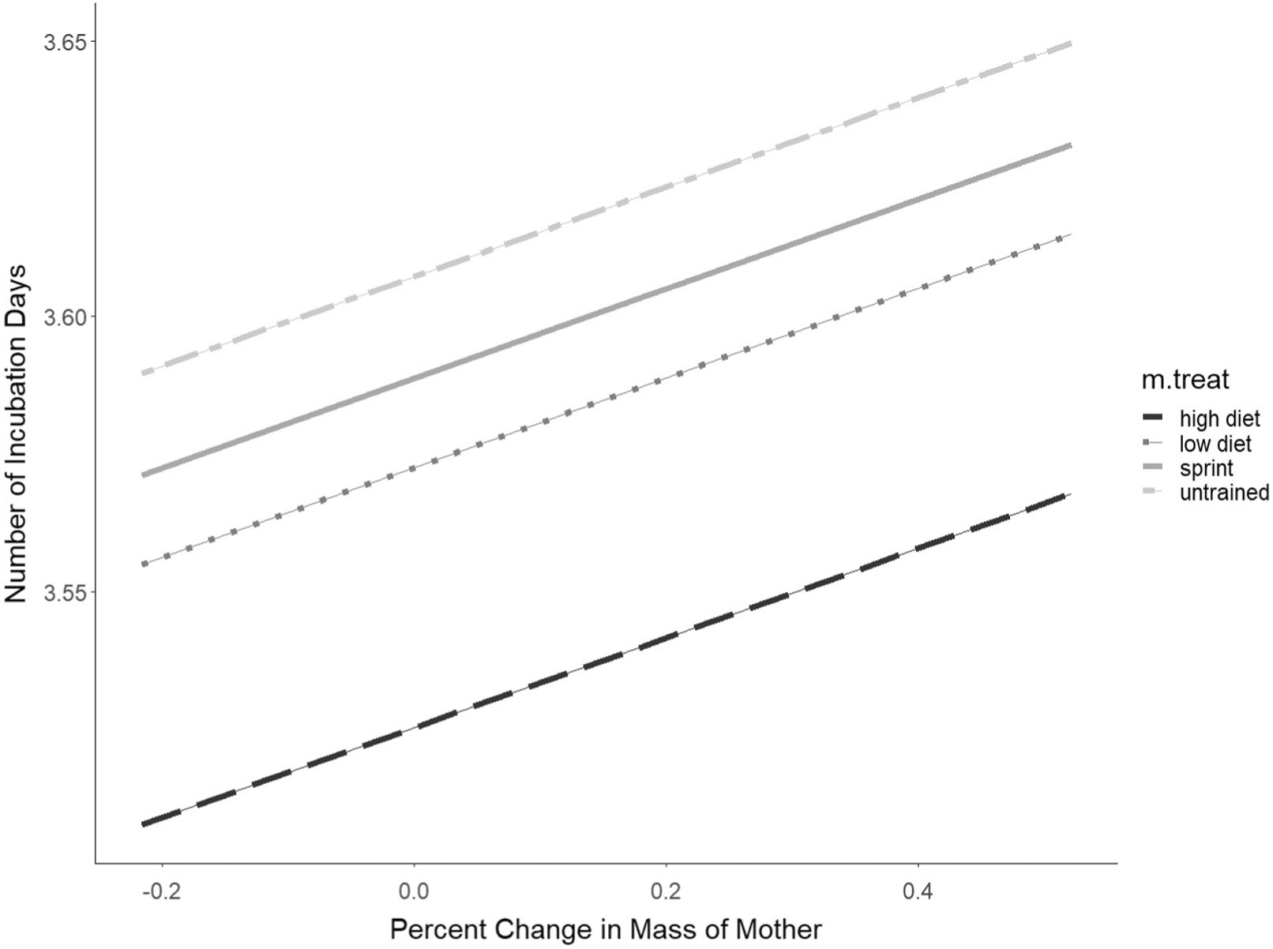
Estimated marginal means for total incubation time when accounting for percent change in body mass.

**TABLE 6:** Best‐fitting models describing the variation in incubation days with percent change in final body mass of the mom as a covariate.

Model term	Coefficient	SE
Intercept	3.53	0.017
Treat (LD)	0.047	0.028
Treat (ST)	0.063	0.024
Treat (UT)	0.082	0.018
%Δm.mass	0.081	0.075

*Note*: The reported coefficients give estimated change in the dependent variable between the baseline category and the categories named in the table (ST = sprint trained, UT = untrained, LD = low diet). Baseline category was the high‐diet group.

### 3.5 Total incubation time

The best model for incubation time retained an effect of percent change in body mass of the mother (Table 1) and treatment on total incubation time (AIC = 527, no. parameters = 2). The next best model contained an interaction between percent change in body mass of the mother and treatment (AIC = 533, no. parameters = 3). The total incubation time for the offspring from the sprint trained (ST) and untrained (UT) groups was significantly longer than that of the high‐diet (HD) group (Figure 1). Incubation times of offspring from the low‐diet (LD) group did not differ significantly from the HD group.

## 4 DISCUSSION

Understanding the factors driving maternal effects is vital if we are to learn how mothers can best prepare their offspring for current environmental conditions. In this experiment, we compared egg and offspring characteristics from female green anoles that were either diet restricted or sprint trained. We tested specific predictions to determine how these different environmental pressures affected their offspring. We incorporated body mass into our analyses because of the known allometric effects of maternal body mass on offspring size (Kindsvater et al., 2012; Sakai & Harada, 2001). Additionally, we refer to “energetic state” or “energetic environment” of the mother throughout as this terminology acknowledges that the changes implemented by the treatments affect the amount of available and allocable energy (Marks et al., 2021, 2022).

Our first prediction that the LD and ST animals would lay significantly fewer eggs than the HD group was supported (Figure 1; Table 1), but the prediction that LD and ST would lay less eggs than the UT group was not. Percent change in mass of the mother was included in the final model and there was a negative correlation between egg number and percent change in maternal mass. The quintessential life‐history trade‐off is between survival and reproduction, which diet restriction is known to promote (Chapman & Partridge, 1996; Mair & Dillin, 2008; Moatt et al., 2016; Regan et al., 2020). Females will forgo current reproduction and extend their lifespan to wait for an environment with more suitable resources (Regan et al., 2020; Stearns, 1989; Sultanova et al., 2021; Thompson, 2013). Our results here are consistent with these earlier results, in that limiting available resources resulted in decreased reproductive rate in female green anoles. However, we also found a difference between these two treatments, in that the ST group laid significantly more eggs than the LD group. It could be that the physiological changes wrought by sprint training are less energetically taxing than diet restriction is to green anoles; indeed, Lailvaux et al. (2018) found that sprint training reduces resting metabolic rates in green anoles, which may result in more energetic resources being available for allocation to reproduction in sprint‐trained mothers. Previous studies manipulating both diet and clutch size have shown that female zebra finches who lay more eggs experience increased muscle atrophy that negatively affects their flight performance, pointing to a key trade‐off between fecundity and performance (Veasey et al., 2000, 2001). Our finding here that sprint training, which is known to increase muscle mass in green anoles, corresponds to an increase in reproductive output is therefore unexpected. To our knowledge, no other study has tested the effects of maternal sprint training on offspring phenotypes in reptiles, which limits our ability to place this finding within a proper comparative context.

Our second prediction that egg mass would be lower within the ST and UT group was not supported when the maternal mass at oviposition was included in the model (Figure 2b; Table 3). Within this model, there is a strong positive correlation between egg mass and mass of the mother at oviposition, and the HD group laid the lightest eggs compared to the other treatments. When looking at the model with %Δm.mass (Figure 2a; Table 2), there is an interaction between body mass of the mother and treatment where the LD and ST lizards have a negative relationship between %Δm.mass and egg mass, while the HD and UT retain the positive relationship. Based on Figure 2, the treatment lizards in a negative energetic environment (i.e., those that lost weight) may have invested energy into laying larger eggs rather than more eggs. These results follow the principles of the bet‐hedging model, whereby females will lay fewer eggs in order to invest more energy into individual offspring (Mitchell et al., 2018; Nussbaum, 1981; Reznick & Yang, 1993; Seger & Brockman, 1987). For example, Mitchell et al. (2018) captured brown anoles at multiple time points throughout a breeding season and found that groups caught later in the season laid fewer eggs but invested more resources into each egg to produce larger offspring.

The mass of the offspring at time of hatching was affected by treatment; but, our third prediction that offspring from the LD and ST lizards would weigh less than those from the UT and HD moms was unsupported. Although year was controlled for via inclusion as a random factor in our model, there is nonetheless a significant difference in hatchling mass between the UT and ST groups and the HD and LD groups (Figure 3; Table 4), such that the offspring from the diet experiment were significantly heavier at hatching. Because of the known allometric effect of maternal body size on offspring body size (Kindsvater et al., 2012; Sakai & Harada, 2001), we also included percent change in mass of the mothers as a covariate to control for any differences in maternal body mass between the experiments. However, this metric was not significant here and was therefore omitted from the final model. A potential mechanism underlying the differences in offspring phenotype between the treatments could be the insulin/insulin‐like signaling network (IIS). This is a highly conserved pathway and its main roles are to facilitate cell growth and division and aspects related to reproduction and metabolism (Duan et al., 2010; Regan et al., 2020; Schwartz & Bronikowski, 2016). Altering maternal environment affects hormones within the insulin/insulin‐like signaling (IIS) network, specifically hepatic expression of insulin‐like growth factor 1 (IGF1) and insulin‐like growth factor 2 (IGF2; Marks et al., 2021, 2022; Regan et al., 2020). The offspring tested within this experiment are derived from two larger, prior experiments where we measured *IGF* expression and showed that diet restriction affects *IGF1* and *IGF2* expression (Marks et al., 2021), and that sprint training also affects *IGF1* and *IGF2* but in a different manner than diet restriction (Marks et al., 2022). It could also be that the difference in hatchling mass, and ultimately incubation period, is due to the increase in experimenter handling time experienced by the mothers in the sprint training experiment. This handling time may have affected corticosterone levels which can affect growth trajectories (Vercken et al., 2007). Although this relationship has not been tested in green anoles (Husak et al., 2015), we know that corticosterone levels were comparable between handled and sprint‐trained yellow‐bellied water skink (*Eulamprus heatwolei*; Langkilde & Shine, 2006). Alternatively, this handling time may have affected maternal IGF expression within these mothers which could impact IGF, and ultimately phenotype, of the offspring. Although this experiment was not designed to explicitly test the link between maternal IGF expression and offspring phenotype, it would be a logical next step to test this relationship.

We made the null prediction (P4) that SVL would not differ between the ST and UT lizards. Offspring from the ST lizards had significantly longer SVLs than those from the UT moms (Figure 4; Table 5), yet the average mass between treatments was not different (Figure 3; Table 4), suggesting that ST offspring potentially allocated energy to bone growth. Differentiation of mesenchymal stem cells occurs during development, producing cells that facilitate bone, muscle, and fat growth (Du et al., 2011; Lanham et al., 2010; Sanger et al., 2012). Variation in maternal environmental conditions in pigs and cattle, such as low nutrient availability, leads to differences in mesenchymal cell differentiation in their offspring (Du et al., 2010). Maternal sprint training may also induce differences in mesenchymal cell differentiation and these differences may manifest themselves by supporting skeletal growth in offspring. It is also of note that limb length is known to be plastic in juvenile *A. carolinensis* (Kolbe & Losos, 2005); consequently, malleability in other skeletal elements of young green anoles cannot be ruled out, although the factor inducing such plasticity here is different. In any case, the proposed mechanism of maternal effects inducing skeletal plasticity via mesenchymal cell differentiation is testable.

The significant difference between experiments seen in hatchling mass was also seen when testing incubation period. Our fifth prediction that incubation period within the LD and ST groups would be higher than the controls (Figure 5; Table 6) was not supported. Outside of questions focusing on incubation temperature, egg phenotype is rarely studied within the context of maternal effects in vertebrates. Development time is, however, commonly measured in insects because of its clear effects on offspring phenotype. For example, development time can be affected by maternal diet in large milkweed bugs (*Oncopeltus fasciatus*), and offspring reared on different diets than their mothers had longer developmental times than siblings reared on the same host plant as their mother (Newcombe et al., 2015; see also Lailvaux et al., 2017). Although our results showed that there was no effect of sprint training or diet restriction on offspring incubation time when compared to their respective control situations (Figure 5; Table 6), we did see a difference between the two experiments. Year was included as a random effect in our final model so it is possible that this difference is due, again, to significantly longer handling time within the sprint training experiment. These effects of the maternal environment on lizard egg phenotypes are seldom explored in reptiles and deserving of more attention.

Maternal effects can be key for animals to best prepare their offspring for the environment in which they are being born (Mousseau & Fox, 1998; Wolf & Wade, 2009). Our hypothesis that maternal dietary restriction and sprint training would have different consequences for the offspring phenotype in green anoles was supported. Our results show that offspring phenotype changes depending on the energetic environment of the mother and the manner in which the energetic environment is imposed. These results highlight an important point that ecologically relevant tasks such as locomotion deserve more attention within the context of maternal effects as they clearly impact offspring phenotype, although the adaptive value of these effects (if any) remains to be seen.

## AUTHOR CONTRIBUTIONS

**Mahaut Sorlin:** Investigation (supporting); writing – review and editing (supporting). **Simon P. Lailvaux:** Conceptualization (supporting); data curation (equal); formal analysis (supporting); funding acquisition (lead); investigation (supporting); methodology (supporting); project administration (supporting); resources (lead); supervision (equal); writing – original draft (supporting); writing – review and editing (supporting). **Jamie R. Marks:** Conceptualization (lead); data curation (lead); formal analysis (lead); investigation (lead); resources (supporting); writing – original draft (lead); writing – review and editing (lead).

## Data Availability

The data that support the findings of this study will be available in Dryad upon acceptance: https://doi.org/10.5061/dryad.3tx95x6kc.
